# Development and evaluation of a fish feed mixture containing the probiotic *Lactiplantibacillus plantarum* prepared using an innovative pellet coating method

**DOI:** 10.3389/fvets.2023.1196884

**Published:** 2023-06-12

**Authors:** Natália Chomová, Sylvie Pavloková, Miriam Sondorová, Dagmar Mudroňová, Adriána Fečkaninová, Peter Popelka, Jana Koščová, Rudolf Žitňan, Aleš Franc

**Affiliations:** ^1^Department of Microbiology and Immunology, University of Veterinary Medicine and Pharmacy in Košice, Košice, Slovakia; ^2^Department of Pharmaceutical Technology, Faculty of Pharmacy, Masaryk University, Brno, Czechia; ^3^Department of Pharmaceutical Technology, Pharmacognosy and Botany, University of Veterinary Medicine and Pharmacy in Košice, Košice, Slovakia; ^4^Department of Food Hygiene, Technology and Safety, University of Veterinary Medicine and Pharmacy in Košice, Košice, Slovakia; ^5^Research Institute for Animal Production, National Agricultural and Food Center, Nitra, Slovakia

**Keywords:** probiotic feed, physical characteristics, viability, nutritional composition, aquaculture

## Abstract

**Introduction:**

Due to the intensification of fish farming and the associated spread of antimicrobial resistance among animals and humans, it is necessary to discover new alternatives in the therapy and prophylaxis of diseases. Probiotics appear to be promising candidates because of their ability to stimulate immune responses and suppress the growth of pathogens.

**Methods:**

The aim of this study was to prepare fish feed mixtures with various compositions and, based on their physical characteristics (sphericity, flow rate, density, hardness, friability, and loss on drying), choose the most suitable one for coating with the selected probiotic strain *Lactobacillus plantarum* R2 Biocenol™ CCM 8674 (new nom. *Lactiplantibacillus plantarum*). The probiotic strain was examined through sequence analysis for the presence of plantaricin- related genes. An invented coating technology based on a dry coating with colloidal silica followed by starch hydrogel containing *L. plantarum* was applied to pellets and tested for the viability of probiotics during an 11-month period at different temperatures (4°C and 22°C). The release kinetics of probiotics in artificial gastric juice and in water (pH = 2 and pH = 7) were also determined. Chemical and nutritional analyses were conducted for comparison of the quality of the control and coated pellets.

**Results and discussion:**

The results showed a gradual and sufficient release of probiotics for a 24-hour period, from 10^4^ CFU at 10 mi up to 10^6^ at the end of measurement in both environments. The number of living probiotic bacteria was stable during the whole storage period at 4°C (10^8^), and no significant decrease in living probiotic bacteria was observed. Sanger sequencing revealed the presence of plantaricin A and plantaricin EF. Chemical analysis revealed an increase in multiple nutrients compared to the uncoated cores. These findings disclose that the invented coating method with a selected probiotic strain improved nutrient composition and did not worsen any of the physical characteristics of pellets. Applied probiotics are also gradually released into the environment and have a high survival rate when stored at 4°C for a long period of time. The outputs of this study confirm the potential of prepared and tested probiotic fish mixtures for future use in *in vivo* experiments and in fish farms for the prevention of infectious diseases.

## 1. Introduction

Aquaculture is an important source of animal proteins throughout the world. The increasing demand for fish and the limited supply of natural habitats necessitate the need to enhance fish farming practices to meet the demand (1). A widespread problem associated with the intensification of animal production is the occurrence of diseases. The solution to this issue is often the massive use of antibiotics, whether in the prevention or treatment of diseases (2, 3). However, this trend causes a rapid increase in the emergence of bacterial resistance, not only in animal production but also in the human population. For this reason, it is essential to discover an alternative solution to the prophylaxis and therapy of diseases (4–6). Promising candidates are immunostimulants of natural origin, especially probiotics, which are also environmentally friendly (7, 8). Probiotics have been used in aquaculture for a long time, but in recent years, they have also become an integral part of improving the growth and resistance of aquatic organisms to various diseases (9). They stabilize and control microbial populations, maintain stable water quality parameters, prevent bacterial and viral infections, improve the quality of feed, support the growth of aquatic organisms by producing vitamins, minerals, and nucleic acids, and increase the survival of aquatic organisms (10). The most common way to select probiotics is through *in vitro* antagonism assays, in which pathogens are exposed to potential probiotics or their extracellular products in a liquid or solid medium, and their growth is monitored (11). In addition, it is essential to know the origin of the probiotics themselves, their safety, and their ability to survive in the gastrointestinal tract of the host to resist bile, low pH, and proteases (2). One of the main criteria for selecting probiotics is their ability to colonize, which means effectively adhering to the intestinal epithelium to limit or prevent intestinal colonization by pathogens (4). Potential probiotics must also have beneficial effects on the host, for example, by stimulating of the immune response (7). One of the promising microbes is *Lactobacillus plantarum* (new nom. *Lactiplantibacillus plantarum* [R2 Biocenol™ CCM 8674)], which was isolated from the intestinal contents of rainbow trout (*Oncorhynchus mykiss*) bred on the Slovak farm Rybárstvo PoŽehy sro. and selected based on the selection criteria for probiotic bacteria. This bacterium is considered to meet all the above criteria for a suitable probiotic. The tested strain demonstrated good growth properties, the ability to survive in the conditions of the fish digestive tract, antagonistic activity against severe bacterial fish pathogens, and susceptibility to antibiotics based on EFSA regulation (9). It also showed a suitable anti-inflammatory response in the trout intestinal cells infected with one of the most serious salmonid pathogens, *Aeromonas salmonicida*. After infection of the intestinal cells with the immunosuppressive pathogen *Yersinia ruckeri, L. plantarum* R2 stimulated the immune response (10). The anti-inflammatory effect of the strain R2 was also confirmed in an experiment on Atlantic salmon, in which enteritis was induced by feeding a pro-inflammatory feed with a high content of soybean flour. A significant reduction in the manifestations of enteritis was observed in salmon that received probiotic feed containing *L. plantarum* R2 and *L. fermentum* R3 (12).

The delivery method of probiotics should be suitable and tailored to the specific animal species and the environment in which they are present. Bacterial suspension alone may not be attractive to fish, which is why probiotics are often used in the form of feed premixes (13, 14). This way, it is possible to administer the exact dose based on the species, number, and size of the fish (15, 16). Natural resources (such as maize flour, milled rice, and soya flour), fillers (such as maltodextrins, lactose, and microcrystalline cellulose), as well as binders (such as various types of starches, alginates, agar, gelatin, chitosan, and various cellulose derivatives, which also increase stability), are used for the formulation of various forms of feed premixes. The most common steps in their preparation include grinding of components, mixing, subsequent granulation, extrusion, drying or cooling, and for longer-term storage, technology aimed at reducing water content and fat stabilization (15). Moreover, it is necessary that the resulting feed premix containing the probiotics be stable because, over time, the number of living bacteria decreases (17). Recently, the patented coating method has been used to produce feed premixes for aquaculture, where the finished feed pellets are first dry coated with sorbent-like colloidal silica, which creates a hydrophilic surface to which the drug is then applied (18). The active substance can be applied both in dry (19) and liquid (20) form as a dispersion of a suitable polymer (21). The drug adheres to the surface through impregnation mechanisms (22). A suitable mixer, preferably a coating pan, which is commonly used in pharmaceutical technology, can be used to apply the coating dispersion to the pellets (18). Since the whole pellet is not moistened here, only its surface, and the amount of water is low, it is possible to apply gentle drying at a low temperature, which will ensure the suitable viability of bacteria (23)^.^ This method is relatively simple, does not require expensive equipment, and makes it possible to produce large batches directly in individual fisheries (24).

The aim of this study was to find a suitable composition for this coating technology for the preparation of feed premix containing *L. plantarum* (R2 Biocenol™ CCM 8674). Several coating polymers were tested, and then the commercial fish pellets (Biomare Inicio 918, Denmark) were dry coated with colloidal silica sorbent (Aerosil® 200) at first, and then aqueous polymer dispersions were applied. For the most promising formulation, an aqueous probiotic suspension was added to the coating dispersion. All samples were evaluated for common pharmacopeial parameters such as particle size, weight uniformity, flow properties, and mechanical resistance. Electronic images of the final dosage form were captured. To investigate the presence of genes producing antimicrobial peptides (plantaricines), Sanger sequencing was performed. The number of live probiotic bacteria was determined after the preparation of probiotic feed and then every month during an 11-month period. Viability was evaluated at two different storage temperatures for probiotic premix (4°C and 22°C). Moreover, probiotic samples were established for *in vitro* release kinetics of probiotic bacteria in water and in a stomach-like environment. Cores and probiotic pellets were also submitted for chemical analysis for composition and nutrient content. This experiment was focused on the preparation of various feed mixtures and the selection of the most suitable one based on physical characteristics for probiotic coating. Evaluation of the survival rate of probiotics during an 11-month period at two different temperatures, the release of probiotics into the water and gastric-like environment, nutrient and chemical analysis, and other *in vitro* methods were performed for confirmation of the efficacy and quality of prepared probiotic pellets, which could be used in the future for *in vivo* experiments and in aquaculture for prevention and treatment of problematic diseases. To date, samples of the final formulation have been used for preliminary clinical trials (25). Subsequently, an extensive clinical study was carried out on large production batches, the results of which will be the subject of a separate article.

## 2. Material and methods

### 2.1. Preparation of screening samples

According to Table 1 (PART I.), aqueous dispersions of the coating polymers were prepared. The polymers used included methacrylate copolymer trademark Eudragit® E 100 (Evonik Industries AG, Germany) combined with citric acid monohydrate (Dr. Kulich Pharma, Czech Republic) in a weight ratio of 3:1. Additionally, soluble corn starch with the trademark Starch 1500® (Colorcon Ltd., USA) was utilized. Finally, polyethylene glycol PEG 6 000 (Merck, USA) was also included in the mixture. The quantities were weighed to achieve concentrations of 10 and 20% in water, respectively. However, due to its high viscosity, only a 10% dispersion of starch could be prepared. The solid substances were first weighed and then added to water in suitable vessels while being stirred on an electromagnetic stirrer MS-PB (DLAB Scientific Inc., China). The dispersions were mixed until a clear (samples E and P) or colloidal (samples S) solution was formed. The mixing time should not be less than 60 min. Furthermore, nine 150 ml wide-necked glass bottles were used, and 50.0 g of fish pellets with a diameter of 2 mm (Biomare Inicio 918, Denmark) were put into into each bottle. Following that, 0.5 g and 1.0 g of freshly sieved Aerosil® 200 (Evonik Industries AG, Germany) with a mesh size of 315 microns were respectively added to the bottles. The bottles were closed, attached to a blender Turbula T2C (Willy A. Bachofen AG Maschinenfabrik, Switzerland), and mixed for 5 min at 40 rpm to form a hydrophilic dry coat on their surface. Subsequently, 5.0 g and 10.0 g (referred to as sample S_1_) of the aqueous coating dispersion were added to the bottles using a pipette. The samples were then mixed again under identical conditions, ensuring the even distribution of the dispersions on the surface of the pellets. The samples were then dried for 4 h at 35 ^o^C in a hot air oven (UF 30, Memmert GmbH, Germany). The dried samples were evaluated for several parameters (see Chapter 2.3). Sample S_3_ was selected for the preparation of coated pellets containing probiotics.

**Table 1:** Composition of samples.

**Sample**	**Pellets**	**Aerosil® 200**	**Eudragit® E 100** ^**a**^		**Starch 1500®**	**PEG 6000**	
			**10%** ^b^	**20%** ^b^	**10%** ^b^	**10%** ^b^	**20%** ^b^
***All components are given in grams***							
**PART I. Screening samples**							
E_1_	50.0	1.0		5.0			
E_2_	50.0	0.5	5.0				
E_3_	50.0	1.0	5.0				
S_1_	50.0	0.5			10.0		
S_2_	50.0	1.0			5.0		
S_3_	50.0	0.5			5.0		
P_1_	50.0	1.0				5.0	
P_2_	50.0	0.5					5.0
P_3_	50.0	1.0					5.0
**PART II. Probiotic sample**							
S_3_L	50.0	0.5			LD^c^		

a With citric acid monohydrate in weight ratio 3:1;

b dispersion in water;

c LD (Lactiplantibacillus dispersion) = 6 g of 25% *L. plantarum* R2 and 0.5 g of Starch 1500®.

### 2.2. Preparation of probiotic samples

The study was conducted using the strain *Lactiplantibacillus plantarum* [new nom. *Lactiplantibacillus plantarum* (R2 Biocenol™ CCM 8674)] stored in the Czech Collection of Microorganisms (CCM) of Masaryk University (Brno, Czech Republic). The characteristics of the strain have been described above, and pellets were prepared according to the already published methodology (23). An 18-hour bacterial culture in 1 L MRS agar (HiMedia, India) was prepared at 37° C on a shaker (C2 Platform Shaker, New Brunswick Scientific, Edison, NJ, USA), which was then centrifuged (4 500 rpm for 15 min) at 22°C on a centrifuge Rotina 420 R (Hettisch, Germany). The supernatant was removed, and the sediment was shaken two times with saline and centrifuged (4 000 rpm for 15 min). The supernatant was discarded, and saline was then added in such an amount as to achieve a 25% v/v dispersion. To 6.0 g of this dispersion, 0.5 g of Starch 1500® (equivalent to the amount used in S_3)_ was added. The mixture was then stirred using an electromagnetic stirrer for 60 min (see Table 1, PART II.). The procedure for coating and drying the samples was analogous to that in Chapter 2.1. Pellets were subjected to the examination of morphology employing scanning electron microscopy (SEM; MIRA3, Tescan Orsay Holding, Czech Republic) to visualize the surface and differences in its morphology (26, 27).

### 2.3. Molecular screening of the presence of plantaricin-related genes

The presence of plantaricin-related genes was investigated in strain *L. plantarum* R2 by PCR using specific primer pairs. *L. plantarum* R2 was cultured on MRS agar at 37°C for 48 h under anaerobic conditions (GasPak system, Becton Dickinson, San Diego, CA, USA). Subsequently, DNA was isolated from the pure bacterial culture using the Quick-DNA Fecal/Soil Microbe Miniprep Kit (Zymo Research, Irvine, USA). The DNA purity and concentration were evaluated by spectrophotometry using NanoDrop 1000 (Thermo Fisher Scientific, Waltham, MA, USA). The PCR reaction was carried out in a thermocycler TProfesional Basic (Biometra GmbH, Gôttingen, Germany), and the mix composition of each reaction consisted of 2 μL (100 ng) of DNA template, OneTaq 2X Master Mix with standard buffer (New England Biolabs, Foster City, CA, USA), molecular water, and primers at a concentration of 33 μM. Amplification of DNA was performed in a total reaction volume of 51 μL. An equal volume of molecular water was used instead of the DNA template as a negative control. The PCR conditions and sequences of each primer pair are listed in Table 2.

**Table 2:** Characteristics of PCR and primer sequences used for the screening of the presence of plantaricin-related genes.

**Bacteriocin** **(target gene)**	**Primer sequence (5′to 3′)**	**PCR conditions**	**Length (bp)**	**Source**
plantaricin A (*plnA*)	GTACAGTACTAATGGGAG CTTACGCCATCTATACG	94°C 2 min, 30 × [94°C 15 s, 55°C 30 s, 72°C 2 min], 72°C 4 min	450	(28, 29)
plantaricin EF (*plnEF*)	GGCATAGTTAAAATTCCCCCC CAGGTTGCCGCAAAAAAAG	95°C 5 min, 30 × [95°C 1 min, 53°C 1 min, 72°C 1 min], 72°C 7 min	428	(30)
plantaricin S (*plnS*)	GCCTTACCAGCGTAATGCCC CTGGTGATGCAATCGTTAGTT	94°C 3 min, 30 × [94°C 1 min, 60°C 1 min, 72°C 30 s], 72°C 5 min	320	(31)
plantaricin W (*plnW*)	TCACACGAAATATTCCA GGCAAGCGTAAGAAATAAATGAG	94°C 3 min, 30 × [94°C 1 min, 55°C 1 min, 72°C 30 s], 72°C 5 min	165	(31)
plantaricin NC8 (*plnNC8βα*)	GGTCTGCGTATA AGCATCGC AAATTGAACATATGGGTGCTTTAAATTCC	94°C 3 min, 30 × [94°C 1 min, 60°C 1 min, 72°C 30 s], 72°C 5 min	207	(31)

The amplification products were separated through electrophoresis on 2% agarose gels and visualized with GelRed (Biotium, Inc., Hayward, CA, USA) under ultraviolet light (32). A 100 bp DNA ladder (New England Biolabs, Foster City, CA, USA) was used as a marker to determine the sizes of the PCR products. Subsequently, the positive amplification products were sent for Sanger sequencing from both forward and reverse directions (Microsynth, Wien, Austria). All sequences were processed using the software Geneious version 8.0.5 (Biomatters, Auckland, New Zealand), and the search for nucleotide sequence homologies was conducted with the online BLAST (Basic Local Alignment Search Tool) algorithm (https://blast.ncbi.nlm.nih.gov/Blast.cgi, accessed on 14 March 2022).

### 2.4. Samples evaluation (physical characteristics, viability and release of probiotics, nutritional, and chemical composition)

The weighing and assessment of weight uniformity, which indicates any changes related to coatings, were conducted using WHT 1 equipment (Pharma Test, Germany) according to Ph. Eur. [2.9.5.] (33) guidelines. The optical microscope DN 25 Lambda (Intarcho-micro, Czech Republic) with a CCD camera Alphaphot-2 (Nikon, Japan) and image analysis software Leco IA 32 (Leco Instruments, USA) were employed to measure the equivalent diameter, dimensions, and sphericity, which reveal changes based on the number of coatings. These measurements were carried out following the procedures outlined in Ph. Eur [2.9.37.] (33). The flow rate, which indicates changes in flow properties related to dosing, was measured using a flowability funnel ZT (Medipo, Czech Republic) following the guidelines specified in the Ph. Eur. [2.9.16.] (33). Bulk and tapped densities and Carr Index with Hausner Number, which indicate changes in the volume of the feed mixture during transport and storage, were evaluated using tester SVM 102 (Erweka GmbH, Germany) following the guidelines outlined in the Ph. Eur. [2.9.34.] (33). Pycnometric density, which relates to the density of the pellet core and the coating level, was evaluated using a helium pycnometer Pycnomatic-ATC (Porotec GmbH, Germany) according to the guidelines in the Ph. Eur. [2.9.23.] (33). Hardness, which demonstrates the ability to withstand crushing and is necessary for the assessment of mechanical resistance during the transport of the feed, was evaluated using the C50 hardness tester (Engineering Systems, UK) and measured in accordance with the guidelines specified in Ph. Eur. [2.9.8] (33). Friability, which indicates the quality of the coating level, was evaluated using a friability tester TAR 10 (Erweka GmbH, Germany) according to guidelines in the Ph. Eur. [2.9.7.] (33). The loss of drying was determined using the halogen moisture analyzer HX204 (Mettler Toledo, Switzerland) [*t* = 105°C, AK3 (1 mg/50s)]. The electrostatic charge characterizing the change of electric charge before and after coating was determined using a Faraday cage JCI 150 equipped with a charge measuring unit JCI 178 (Chilworth Technology Ltd., UK) according to an already published method (34). The sample preparation for the microscopic measurement included mounting the specimen on an SEM stub using conductive carbon double-faced adhesive tape (Agar Scientific, Essex, UK), followed by coating under an argon atmosphere with a 20 nm gold layer using the sputter coater (Q150R ES Rotary-Pumped Sputter Coater/Carbon Coater, Quorum Technologies, Laughton, UK). Images were captured with a secondary electron detector at an accelerating 127 voltage of 3 kV. The viability of *L. plantarum* R2 Biocenol™ (CCM 8674) during an 11-month period in different storage temperatures (4°C, 22°C) was evaluated using the plate count method on MRS agar (Biolife, Italy). The release of probiotic bacteria in two different environments (pH = 7 and pH = 2.5) was established using a device (Erweka MDL ZT3-2, Germany). Samples of water and stomach-like solution were tested at multiple time intervals (10 min., 1 hour, 4 hours, 8 hours, and 24 hours). The number of probiotics was also evaluated using the plate count method on MRS agar (Biolife, Italy). The nutritional content and chemical composition were determined for control cores and probiotic pellets. Individual parameters were evaluated in two forms – freshly made cores and the dry matter of these cores. The chemical composition of feed was determined using the Laboratory of Feed Analysis of the Research Institute for Animal Production Nitra, National Agricultural and Food Center. The content of dry matter, crude protein, fiber, fat, and ash was determined according to Commission Regulation (EC) No. 152/2009 (35).

### 2.5. Data analysis

The statistical analysis of the pellet's physicochemical properties was based on principal component analysis (PCA), which was employed as a suitable technique for assessing multivariate data. PCA primarily aimed for a quick and comprehensive visual assessment of the interrelationships among the pellet samples and/or their characteristics via two approaches. The first PCA model was built to assess screening samples and compare them with uncoated pellets (cores) (*PCA model 1* in the Section 3). The second PCA model included all samples and was used for the comparison of pellets with (S_3_L) and without probiotics (screening samples) (*PCA model 2*). The PCA results were visualized utilizing the PCA scores plot and PCA loadings plot, which enable the observation of mutual similarity/difference between samples based on selected measured properties (weight uniformity, equivalent diameter, sphericity, flow rate, Hausner ratio, hardness, and friability). For data processing, software R, version 3.4.4, was used (36). The results of *in vitro* release of probiotic bacteria and the viability of probiotic strain were statistically evaluated in the program GraphPad Prism using a two-way ANOVA analysis of variance. Two different additional tests were used: Tukey's *post-hoc* test for the release of probiotics and Dunnett's test for viability. The results were evaluated at statistically significant levels: *p* < 0.05, 0.01, and 0.001.

## 3. Results and discussion

### 3.1. Mechanisms of the coating

The satisfactory properties of the coated pellets were achieved using a special technology of dry coating with colloidal silicon dioxide. A hydrophilic layer was firmly attached to the surface of the oleophilic pellets. Subsequently, in the next step, a starch layer containing probiotics was applied to this hydrophilic layer. The principle of dry coating is that strongly negatively charged colloidal silica (Aerosil® 200) is electrostatically bonded to a slightly positively charged core, thereby neutralizing the charge and forming a tightly bound hydrophilic coating layer that allows adhesion of the hydrophilic starch layer. The individual values of the electric charge of the cores, colloidal silica, pellets with colloidal silica, the sample with the subsequent starch coating S_3_, and finally, the sample with the probiotic S_3_L are shown in the following Table 3.

**Table 3:** Surface charge of intermediates and final samples.

**Sample**	**Charge (nC/g)**
Cores	0.025 ± 0.021
Aerosil® 200	−4.668 ± 0.669
Cores coated by Aerosil® 200	−0.026 ± 0.012
S_3_	0.029 ± 0.010
S_3_L	0.021 ± 0.003

**Figure 1:**
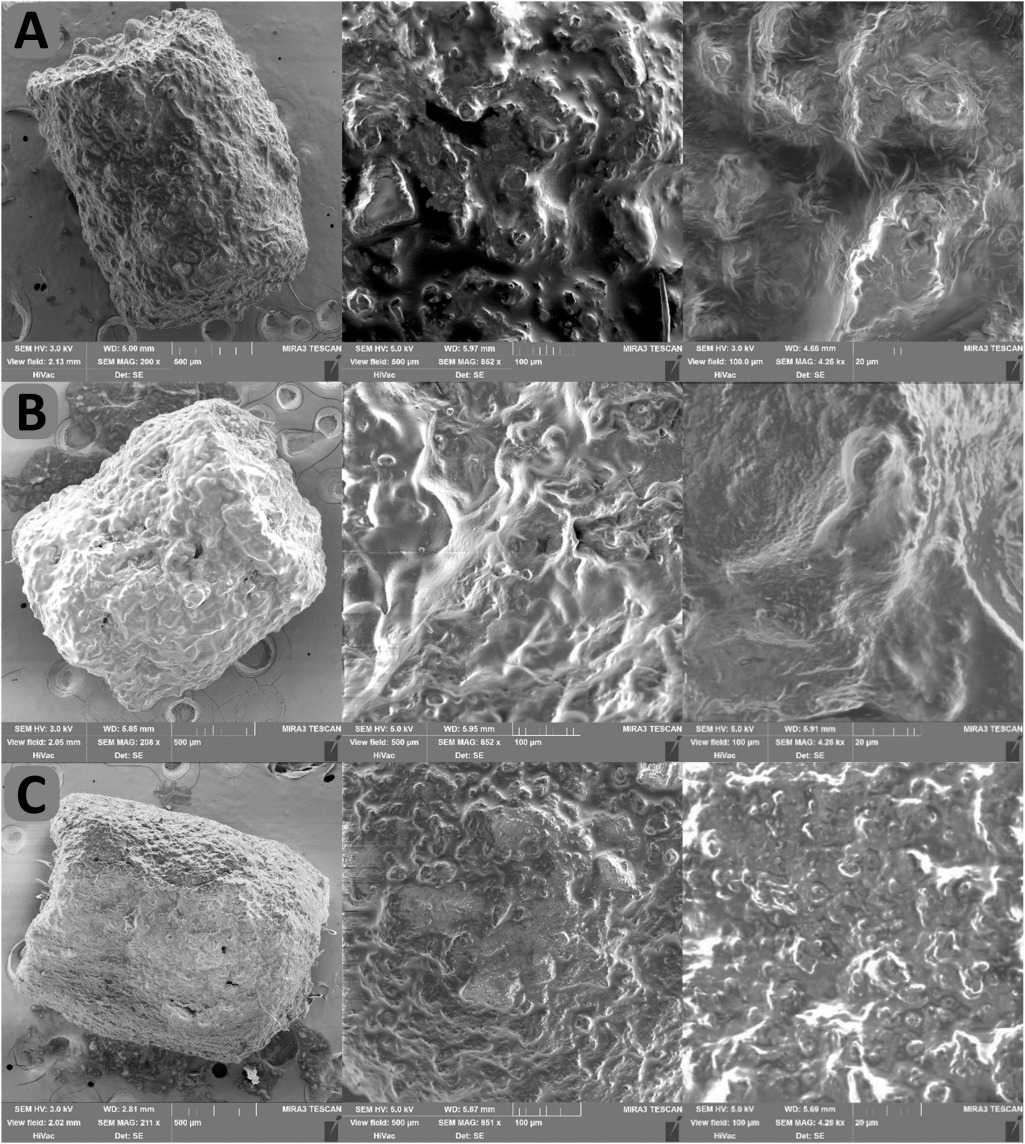
SEM images of **(A)** cores, **(B)** cores coated by Aerosil® 200, and **(C)** Sample S3L.

The coating process was examined through SEM on uncoated cores (Figure 1A), cores dry coated with Aerosil® 200 (Figure 1B), and subsequently with probiotic dispersion (Figure 1C). This analysis allows us to comprehend differences in morphology and structure between pellets coated with various technologies and substances (37, 38). We observed roughness and numerous creases on the surface of the cores (picture A). Aerosil® 200 then formed a uniform layer on the surface of the cores, which practically did not change the morphology of the structure (pictures B). This allowed the formation of a layer from probiotic dispersion, which ultimately led to the creation of a smoother and more mechanically resistant surface of the coated pellets (pictures C).

### 3.2. Presence of plantaricin-related genes

Based on the molecular screening of various *pln* genes in *L. plantarum* R2, the presence of *plnA* and *plnEF* genes in this strain was detected. The identities of the amplified *plnA* and *plnEF* genes were further confirmed by DNA sequence analyses, whereas these sequences showed 100% homology with their respective genes. The bactericidal activity of plantaricines is linked with their ability to permeabilize the membranes of pathogenic bacteria. Therefore, these bacteriocins have great potential in the prevention and treatment of diseases, not only in fish breeding (39).

### 3.3. Physical characteristics of pellets

**Figure 2:**
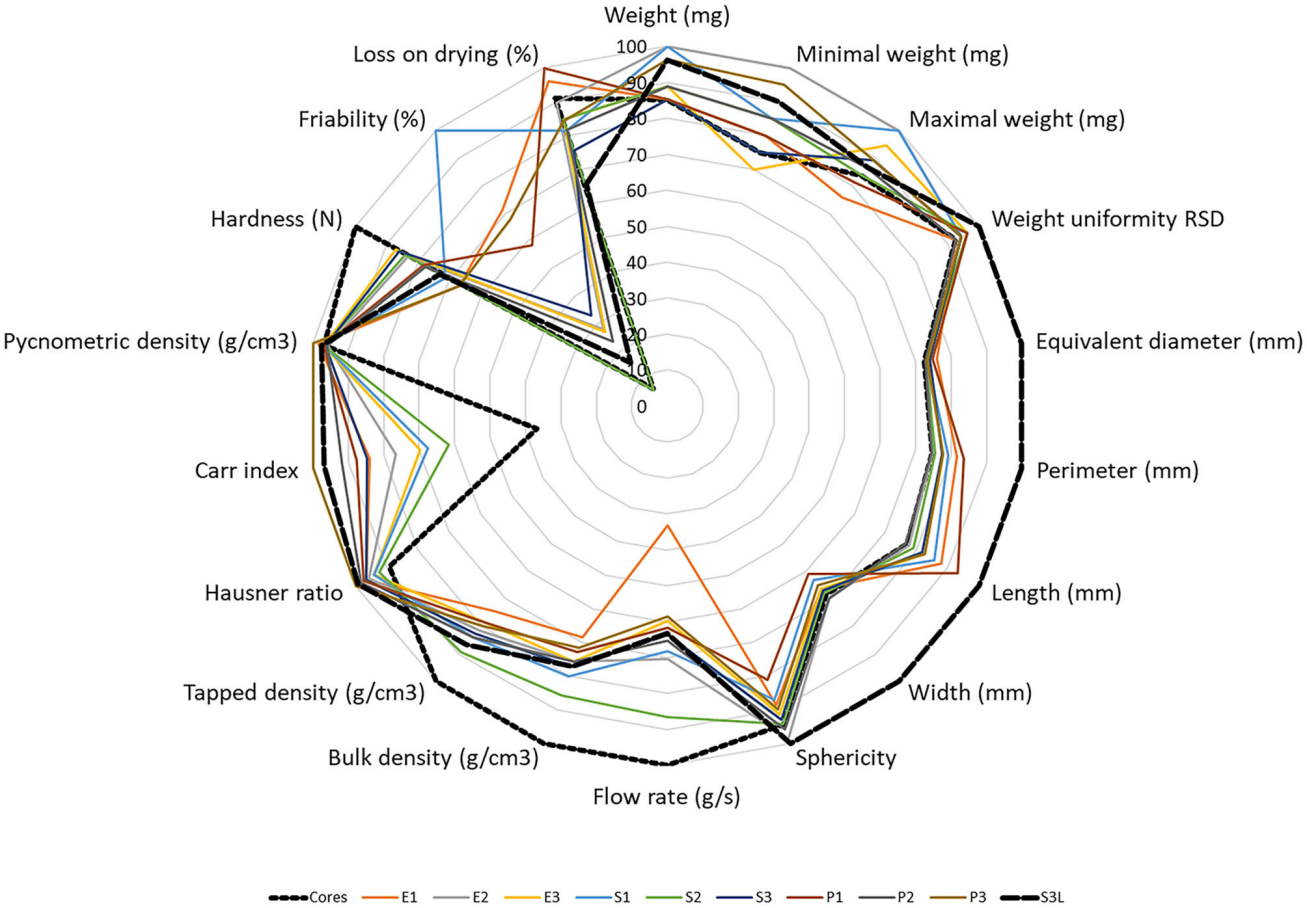
A radar chart converting the values of individual sample quantities into percentages.

Examining the physical properties of the individual samples listed in and proportionally expressed in the radar graph (Figure 2), it is possible to state the morphological similarity of all the prepared samples. Dimensions, densities, and resulting characteristics such as sphericity, Hausner number, Carr index, and hardness were virtually almost indistinguishable from the original feed pellets (cores). Together with the low weight uniformity value, this indicates the formation of a uniform pellet coat in all samples. This is consistent with the previous understanding of coated pellets used for pharmaceutical use (40, 41). Indeed, a significant difference can be observed between the cores themselves and the sample containing the probiotic (S_3_L), which is due to the bacteria content itself. The only variable that is significantly different is friability. Its value increases with the amount of coating. For samples with a lower coating layer content, friability is satisfied, as a value of up to 1.7% is considered acceptable for pellets (42). The fact that the addition of probiotics to the S_3_L sample leads to the lowest friability is encouraging, which indicates the high durability of the coating layer. Therefore, as can be seen from Table 4 and Figure 2, most of the samples show good physical properties, ensuring good flow characteristics of the pellets and their uniform dosage and sufficient mechanical resistance during storage and handling. Therefore, the final choice of composition was determined not only by the resulting physical properties but also by the ease of their preparation. During the coating of individual samples, the pellets of most compositions partially stuck together, and aggregates remained present in the samples after drying. These aggregates were possible under very low mechanical pressure and were easy to separate. The only exception was sample S_3_, where no agglomeration occurred. Therefore, the composition of this sample, in terms of trouble-free preparation, was chosen for further processing. Although Eudragit® E (20, 21), like PEG (15, 43), is commonly used to prepare fish medicated compound feed, starch is also beneficial as a substrate for which a longer survival of probiotics can be expected (44–47).

**Table 4:** Physical characteristics of samples.

**Variable**	n^*^	Sample^**^										
		**Cores**	**E**_1_	**E**_2_	**E**_3_	**S**_1_	**S**_2_	**S**_3_	**P**_1_	**P**_2_	**P**_3_	**S**_3_ **L**
Weight (mg)	20	2.3	2.3	2.7	2.4	2.7	2.4	2.3	2.3	2.4	2.6	2.6
		(0.4)	(0.4)	(0.5)	(0.6)	(0.5)	(0.4)	(0.5)	(0.4)	(0.4)	(0.4)	(0.4)
Minimal weight (mg)	20	1.5	1.6	2	1.4	1.7	1.7	1.5	1.6	1.7	1.9	1.8
Maximal weight (mg)	20	3.1	2.8	3.7	3.5	3.7	3.1	3.3	3	3.2	3.3	3.2
Weight uniformity RSD	20	4.9	4.9	5	5.1	5	5.1	5	5.1	4.9	5	5.3
Equivalent diameter (mm)	100	1.76 (0.12)	1.85 (0.15)	1.79 (0.13)	1.79 (0.17)	1.78 (0.13)	1.77 (0.12)	1.80 (0.16)	1.82 (0.15)	1.77 (0.13)	1.78 (0.14)	2.43 (0.19)
Perimeter (mm)	100	6.94 (0.93)	7.59 (1.08)	6.95 (1.08)	7.22 (1.23)	7.35 (0.97)	7.01 (0.90)	7.20 (1.05)	7.77 (1.17)	6.93 (0.88)	7.23 (1.06)	9.29 (1.15)
Length (mm)	100	2.47 (0.58)	2.82 (0.62)	2.49 (0.61)	2.65 (0.70)	2.75 (0.59)	2.53 (0.53)	2.62 (0.60)	2.99 (0.68)	2.45 (0.54)	2.65 (0.63)	3.21 (0.69)
Width (mm)	100	1.02 (0.19)	0.99 (0.16)	1.04 (0.18)	0.99 (0.18)	0.94 (0.18)	1.01 (0.17)	1.00 (0.18)	0.91 (0.17)	1.04 (0.19)	0.97 (0.19)	1.49 (0.27)
Sphericity	100	0.65 (0.11)	0.61 (0.11)	0.68 (0.13)	0.63 (0.13)	0.60 (0.11)	0.65 (0.11)	0.64 (0.12)	0.56 (0.12)	0.66 (0.11)	0.62 (0.12)	0.69 (0.11)
Flow rate (g/s)	3	78.9	26.2	55.6	47.2	53.8	68.2	50.2	48.6	51.5	46.2	49.9
		(2.8)	(6.4)	(2.4)	(1.1)	(4.2)	(3.1)	(2.8)	(2.1)	(4.3)	(6.5)	(0.9)
Bulk density (g/cm^3^)	1	0.7	0.48	0.53	0.53	0.56	0.6	0.54	0.51	0.53	0.5	0.54
Tapped density (g/cm^3^)	1	0.75	0.56	0.61	0.59	0.63	0.67	0.62	0.59	0.63	0.6	0.65
Hausner ratio	1	1.07	1.16	1.15	1.13	1.13	1.11	1.16	1.17	1.18	1.2	1.19
Carr index	1	6.15	13.98	12.79	11.63	11.25	10.26	14.12	14.61	15.29	16.67	16.13
Pycnometric density (g/cm^3^)	3	1.3037 (0.0014)	1.3262 (0.0027)	1.2900 (0.0009)	1.3063 (0.0037)	1.3065 (0.0018)	1.3010 (0.0041)	1.3002 (0.0015)	1.3063 (0.0021)	1.2910 (0.0019)	1.3465 (0.0053)	1.3150 (0.0013)
Hardness (N)	10	15.6	10.4	13	13.6	11.1	13.1	13.4	12.2	12.1	10.4	11.4
		(2.8)	(3.7)	(3.3)	(3.2)	(3.6)	(2.4)	(2.4)	(2.7)	(2.1)	(3)	(3.4)
Friability (%)	1	0.19	2.21	0.86	0.83	3.1	0.18	1.02	1.81	0.73	2.1	0.49
Loss on drying (%)	1	3.29	3.47	3.24	3.06	2.94	3.06	2.73	3.61	2.95	3.04	2.37

* n – the number of measurements;

** Data in the format: mean value (SD).

**Figure 3:**
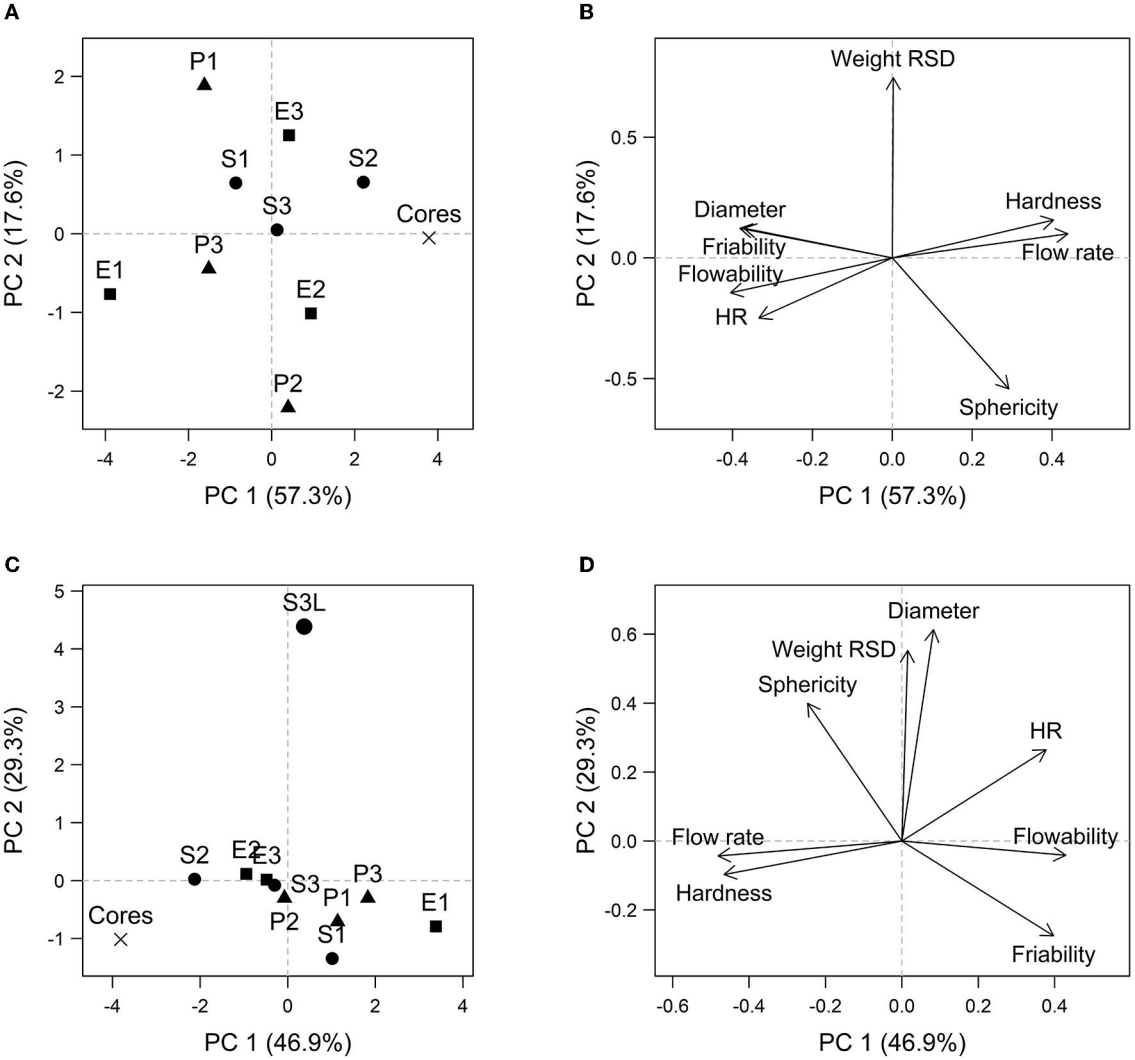
PCA scores plot **(A, C)** and PCA loadings plot **(B, D)** for models 1 (the top) and 2 (the bottom). PCA scores plot comprised screening samples and cores (PCA model 1) or all samples included a probiotic sample (S3L) (PCA model 2). PCA loadings plot included variables/arrows: *Weight RSD*—weight uniformity expressed as RSD of weight, *Diameter*—equivalent diameter, *Sphericity, Flow rate, Flowability, HR*—Hausner ratio, *Hardness*, and *Friability*.

The statistical quantification of the relationship between the composition of the samples and their properties is dealt with by the PCA statistical model presented in Figure 3.

Using PCA model 1, the correlation data structure of the screening sample's physicochemical properties can be assessed. The first two principal components (PCs) described 75.3 % of total variability together.

In the scores plot (Figure 3A), the uniform distribution of samples throughout the ordination space, indicating the absence of clustering samples based on pellet composition, is clearly visible. Sample S3 is located in the center of the coordinate system, i.e., it has “average” properties compared to the other samples. Cores are located in the left part of the graph; thus, they have some extreme properties relative to the screening sample set.

Regarding the monitored pellet properties, the main trend of the explained variability is in the direction of the PC1 axis in the loadings plot (Figure 3B). A positive correlation between hardness and flow rate can be observed in this direction due to the low angle between the corresponding arrows. Hausner ratio and, to a large extent, equivalent diameter and friability are strongly negatively correlated with the previously mentioned quantities since the respective vectors point to the opposite side of the plot. Therefore, samples with larger pellets show higher friability and a deterioration of the flow properties (higher HR and lower flow rate), and hardness, compared to samples of smaller pellet size, can also be observed for these samples. Along the PC2 axis, the samples can be distinguished based on weight uniformity and sphericity. Thus, it can be said that more spherical pellets also have a higher weight uniformity, as the arrow represents the RSD of weight, and the arrow for sphericity is directed approximately against each other.

Based on the direction of the discussed quantity vectors, it can be concluded that samples with ideal physicochemical properties are located in the lower left quadrant of the scores plot, which corresponds to the samples: cores, P2, E2, and then S2 and S3.

The cores differ from the other samples mainly by the value of the quantities correlated with the PC1 axis, i.e., after coating, the resulting pellets are understandably larger, but friability also increases, the pellets have lower hardness, and the flow properties deteriorate to some extent.

Based on PCA model 2, the effect of including a sample coated with probiotic S_3_L in the analysis of the data set's structure is evident. The first two PCs explain 78.2 % of total variability together. The S_3_L sample is distinctly different from pellets without the addition of probiotics (cores and screening samples) in the PC2 axis direction (Figure 3C).

Similar dependencies apply for PCA model 2 and model 1. However, after including the S_3_L sample in the analysis, the correlation between sphericity and weight uniformity changed (Figure 3D). Specifically, an increase in sphericity corresponded to a higher RSD of weight, indicating a decrease in weight uniformity. Moreover, there was a strong correlation between equivalent diameter and RSD of weight, indicating that larger pellets exhibited poorer weight uniformity.

Compared to the other samples from the data set, S_3_L pellets can be characterized mainly by their larger size, lower weight uniformity, higher sphericity, and higher Hausner ratio; other quantities do not play such a considerable role in distinguishing this sample from others.

### 3.4. Viability of probiotic strains and release of probiotics into the environment

**Figure 4:**
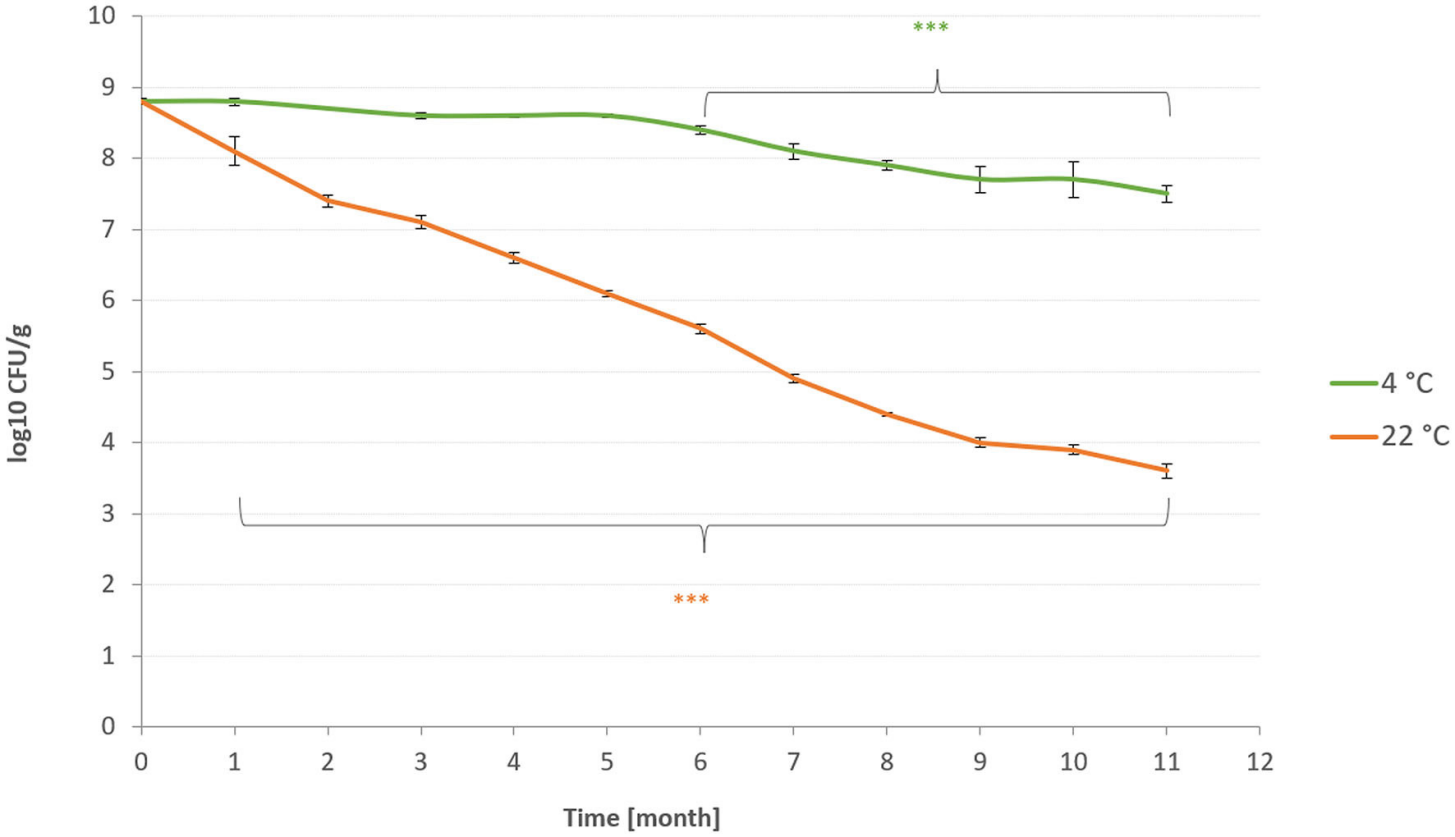
Viability of the *L. plantarum* R2 strain. The * symbol indicates the statistically significant differences between values. Number of stars indicate the level of significance (**p < 0.01, and ***p < 0.001).

The viability of *L.plantarum* R2 Biocenol™ (CCM 8674) was tested to determine optimal conditions for the storage of probiotic feed. Coated pellets (Biomare Inicio 918, Denmark) were observed for an 11-month storage period at different storage temperatures (4°C and 22°C). It was found that the number of *L. plantarum* was maintained almost at the same level for the whole storage period in the case of probiotic pellets stored at 4°C (Figure 4). The first significant loss of probiotic bacteria was noted after six months of storage. However, the observed difference in the quantity of LAB between the first and last months of storage was only one logarithm. On the contrary, it can be seen that at 22°C, the count of probiotic bacteria significantly decreases every month. This phenomenon can be caused by the oxidation of fat in pellets, which may occur at higher temperatures and negatively affect the viability of probiotic strains. We assumed that higher temperatures could also change other pharmaceutical parameters in an unfavorable way and degrade the general quality of the pellets. Based on these results, it can be concluded that probiotic pellets prepared with the invented coating technology should be stored at 4°C to preserve the quality of the pellets and the number of living probiotic bacteria. Storage at higher temperatures should be minimized at inevitable times. Many studies have been published on various coating technologies (biobased dispersions, different drying methods, and the addition of antioxidants) or storage conditions (temperature and pressure) of feed to improve the viability of feed containing probiotics (48–50).

**Figure 5:**
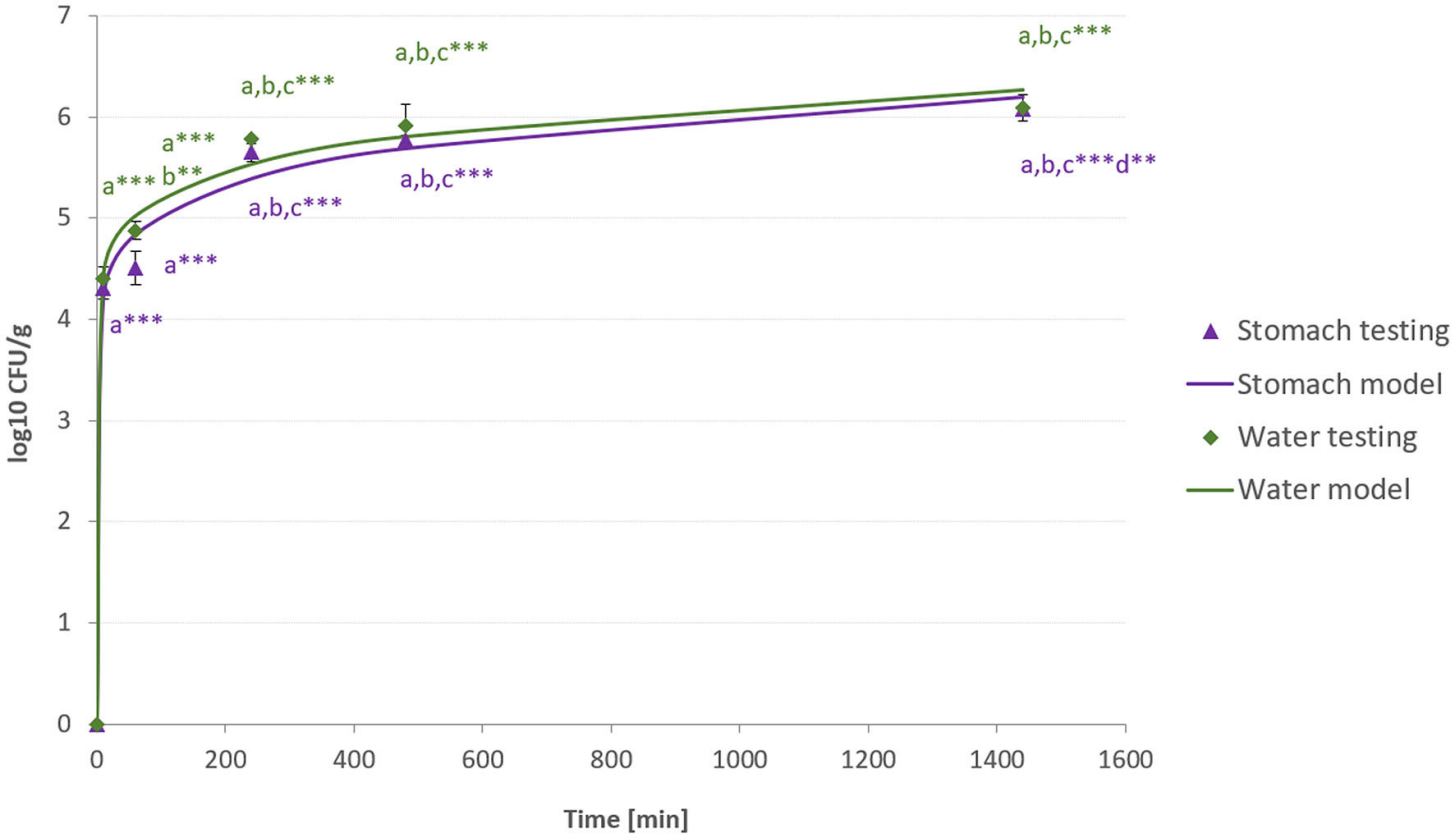
Release of probiotic bacteria in water and a stomach-like environment. The * symbol indicates the statistically significant differences between values. Number of stars indicate the level of significance (**p < 0.01, and ***p < 0.001). a-significant difference compared to time 0 min. b-significant difference compared to time 10 min. c-significant difference compared to time 60 min. d-significant difference compared to time 240 min.

The selected strain for this experiment showed a high level of tolerance to various values of pH, bile, temperature, and suitable growth properties (9). The pH level of the stomach in salmonids is approximately 2. Therefore, the chosen strain has to show resistance against low pH values (51). However, as we can see from other studies, not all lactic acid bacteria have this property (52–54). Fish feed is mostly applied to the water in which fish are located (55, 56). Therefore, it is important to determine how probiotic bacteria are released into the environment and how many bacteria are able to survive both in the intestine and in the water. For this testing, two media were prepared: a stomach-like environment with HCl (pH 2) and water with pH 7. As can be seen in Figure 5, the release of probiotic bacteria from pellets had the same trend in both environments, and there was no bigger difference. Dissolution tested in a gastric-like environment simulates peristalsis in the intestinal tract of fish, so it can be concluded that probiotics can be properly used by fish from eaten pellets. Under normal circumstances, predatory fish such as salmonids consume feed almost immediately from the water's surface. However, the water environment was tested in the case of other fish species, which can be fed in other ways (57). However, the consumption of pellets is very quick for salmonids' potential to use released probiotics from the water. Probiotics from feed can be released into the water from excess feed or excreted feces and improve water quality (58). In the beginning, at the 10-min mark, a significant increase in probiotic levels was detected in both environments, indicating the rapid infiltration of probiotics into these environments. This initial release depends on the structure and composition of pellets, and in farm breeding, this effect is significant and relevant. However, there are studies, especially in human medicine, where an initial time lag and gradual release over several hours are desirable (59). As can be seen from Figure 5, there was a significant increase in released probiotics in both environments in almost every subsequent sampling compared to the previous. It was found that *L. plantarum* was released from pellets even after 24 h. Based on this result, we can conclude that probiotic bacteria are able to survive both in the water and in the gut of fish, where the pH value is low. A great amount of living, released lactic acid bacteria over a long time is probably caused by a combination of Aerosil® 200, which has a high specific surface area and is able to increase the surface area of pellets, whereas starch acts as a prebiotic for *L. plantarum* and more LAB suspension is able to bond up on the surface of pellets. In the end, we can state that the chosen composition and coating technology of probiotic pellets are suitable for use in aquaculture. The significance of methods of incorporating probiotics and proper selection of excipients has already been emphasized in multiple studies (60–62).

### 3.5. Chemical nutrition composition of pellets

Pellet quality and composition are crucial criteria that can improve the digestibility of feed and enhance the health and disease resistance of fish (63–65). As early as 1990, Cho, C.Y., published an article explaining the importance of fundamental components of nutrition with an emphasis on salmonid aquaculture (66). A number of experiments have been conducted in which pellets were enriched with supplements to determine the relationship between the composition of pellets and their influence on fish health (67–71). Our analysis of the chemical composition showed that control cores had a higher content of nitrogenous substances and of the nitrogen-free extract, as well as phosphorus, magnesium, and iron (Table 5). On the contrary, pellets coated with Aerosil 200® and probiotic suspension contained more rough fiber, fat, ash, calcium, and some amino acids (valine, isoleucine, leucine, phenylalanine, lysine, and arginine). This finding is essential because most of the increased amino acids are essential amino acids that fish are not able to synthesize and have to be provided in the feed. Later-formed proteins are necessary for the further growth and proper development of fish (72–74). Ngoh et al. (72) also conducted an experiment where different types of pellets were tested for feeding, and later fish were evaluated for nutrigenomic and nutritional parameters. It was found that groups differed in growth parameters, the morphology of the gut, and also in transcriptomic profiles (72). According to these results, we can state that the composition of feeding pellets can affect the physiology of fish, biochemistry, gene expression of various molecules, and also the quality of meat.

**Table 5:** Chemical and nutrition composition of control and probiotic pellets in an original prepared form and in the dry matter.

**Nutrient content**	**Control cores**			**Probiotic cores**	
	**Unit**	**Fresh core**	**Dry matter**	**Fresh core**	**Dry matter**
Dry matter	g/kg	937.58	1000	868.34	1000
Water	g/kg	62.42		131.66	
N - substances	g/kg	499.96	533.23	439.36	506
Rough fiber	g/kg	9.73	10.38	17.61	20.28
Fat	g/kg	190.36	203.03	194.5	223.99
Ash	g/kg	73.79	78.7	75.09	86.48
Nitrogen-free extract	g/kg	163.76	174.66	141.76	163.26
Organic matter	g/kg	863.79	921.3	793.25	913.52
Total calcium	g/kg	10.08	10.76	10.43	12.02
Total phosphorus	g/kg	12.24	13.06	5.63	6.48
Magnesium	g/kg	4.45	4.74	2.24	2.58
Sodium	g/kg	3.61	3.86	3.5	4.03
Potassium	g/kg	6.83	7.28	6.26	7.21
Iron	mg/kg	359.37	383.29	244.88	282.01
Manganese	mg/kg	19.09	20.36	20.28	23.35
Zinc	mg/kg	98.04	104.57	90.7	104.45
Copper	mg/kg	87.16	92.97	78.4	90.3
Aspartic acid	g/kg		49.19		50.7
Threonine	g/kg		22.3		24.27
Serine	g/kg		25.5		25.88
Glutamic acid	g/kg		88.77		89.28
Proline	g/kg		25.46		25.42
Glycine	g/kg		26.2		26.55
Alanine	g/kg		29.37		28.57
Valin	g/kg		16.62		23.98
Izoleucine	g/kg		10.95		16.53
Leucine	g/kg		38.43		42.94
Tyrosine	g/kg		13.17		15.33
Phenylalanine	g/kg		21.32		24.42
Histidine	g/kg		20.23		18.71
Lysine	g/kg		32.31		36.35
Arginine	g/kg		28.08		31.05
Methionine	g/kg		10.06		9.48
Cystine	g/kg		6.46		5.66

The Blue color illustrates the differences noted between fresh control cores and dry matter of control cores. The Yellow/brown color indicates the differences detected in fresh probiotic pellets and probiotic dry matter against fresh control cores.

## 4. Conclusion

In summary, it can be stated that the invented technology based on the combination of dry coating of pellets with colloidal silicon dioxide followed by coating with a starch hydrogel containing a probiotic creates a mechanically resistant coating on the surface of the oleophilic pellets. Moreover, this process did not worsen pharmaceutical parameters, including the surface and sphericity of feed pellets. *L. plantarum* R2 showed tolerance to low pH levels and a good ability to release into this environment during a 24-hour period. Temperature plays a significant role in the viability of probiotic bacteria, but based on our results, we can say that storage at 4°C is optimal for the maintenance of viability and good quality of pellets, even for long-term storage. Based on Sanger sequencing, the presence of plantaricin-related genes (*plnA* and *plnEF*) in the tested strain was confirmed, which is a prerequisite for their possible production, which must be confirmed at the protein level. In conclusion, we can say that designated coating technology and chosen probiotic bacteria are suitable for the preparation and use of probiotic pellets in *in vivo* experiments and, later, potentially in farm breeding.

## Data availability statement

The original contributions presented in the study are included in the article/supplementary material, further inquiries can be directed to the corresponding author.

## Author contributions

AFr and NC contributed to the conception, design of the study, and wrote the first draft of the manuscript. AFr, SP, NC, MS, RŽ, and DM contributed to the methodology. SP and NC performed the statistical analysis. AFr, NC, SP, MS, AFe, and RŽ participated in the investigation. AFr and DM supervised the process of writing, provided funding, and assisted with project administration. All authors have read and agreed to the published version of the manuscript.

## References

[1] Hines IS Santiago-Morales KD Ferguson CS Clarington J Thompson M Rauschenbach M. Steelhead trout (*Oncorhynchus mykiss)* fed probiotic during the earliest developmental stages have enhanced growth rates and intestinal microbiome bacterial diversity. Front Mar Sci. (2022) 9:1021647. 10.3389/fmars.2022.1021647 PMC11633451 39664599

[2] Kailasapathy K Chin J. Survival and therapeutic potential of probiotic organisms with reference to Lactobacillus acidophilus and Bifidobacterium spp. Immunol. (2000) 78:80–8. 10.1046/j.1440-1711.2000.00886.x 10651933

[3] Hemamalini N Shanmugam S Kathirvelpandian A Eswaran S Agarwal D. A critical review on the antimicrobial resistance, antibiotic residue and metagenomics-assisted antimicrobial resistance gene detection in freshwater aquaculture environment. Aquac. Res. (2022) 53:344–66. 10.1111/are.15601

[4] Dodoo CC Wang J Basid AW Stapleton P Gaisford S. Targeted delivery of probiotics to enhance gastrointestinal stability and intestinal colonization. Int J Pharm. (2017) 530:224–29. 10.1016/j.ijpharm.2017.07.068 28764983

[5] Rohani MF Islam SM Hossain MK Ferdous Z Siddik MA Nuruzzaman M. Probiotics, prebiotics and synbiotics improved the functionality of aquafeed: upgrading growth, reproduction, immunity and disease resistance in fish. Fish Shellfish Immunol. (2021) 120:569–89. 10.1016/j.fsi.2021.12.037 34963656

[6] Caputo A Bondad-Reantaso MG Karunasagar I Hao B Gaunt P Verner-Jeffreys D. Antimicrobial resistance in aquaculture: a global analysis of literature and national action plans. Rev Aquac. (2023) 15:568–78. 10.1111/raq.12741

[7] Wang T Teng K Liu Y Shi W Zhang J Dong E. *Lactobacillus plantarum* PFM 105 promoes intesinal development through modulation of gut microbiota in weaning piglets. Front Microbiol. (2019) 10:90. 10.3389/fmicb.2019.00090 30804899 PMC6371750

[8] Yousuf S Tyagi A Singh R. Probiotic supplementation as an emerging alternative to chemical therapeutics in finfish aquaculture: a review. Probiotics Antimicrob Prot. (2022) 1–18. 10.1007/s12602-022-09971-z 35904730

[9] Feckaninova A Koscova J Mudronova D Schusterova P Cingelova Maruscakova I Popelka P. Characterization of two novel lactic acid bacteria isolated from the intestine of rainbow trout (Oncorhynchus mykiss, Walbaum) in Slovakia. Aquaculture. (2019) 506:294–301. 10.1016/j.aquaculture.2019.03.026

[10] Cingelova Maruscakova I Schusterova P Popelka P Gancarcikova S Csank T Feckaninova A. Effect of autochthonous lactobacilli on immunologically important molecules of rainbow trout after bacterial infection studied on intestinal primoculture. Fish Shellfish Immunol. (2021) 119:379–83. 10.1016/j.fsi.2021.10.021 34687878

[11] Fijan S Frauwallner A Langerholc T Krebs B Younes JATHN Heschl A. Efficacy of using probiotics with antagonistic activity against pathogens of wound infections: an integrative review of literature. BioMed Res Int. (2019) 11:486. 10.1155/2019/7585486 31915703 PMC6930797

[12] Nimalan N Sørensen SL Feckaninova A Koscova J Mudronova D. Mucosal barrier status in Atlantic salmon fed marine or plant-based diets supplemented with probiotics. Aquaculture. (2022) 47:737516. 10.1016/j.aquaculture.2021.737516

[13] Irianto A Austin B. Probiotics in aquaculture. J Fish Dis. (2002) 25:633–42. 10.1046/j.1365-2761.2002.00422.x

[14] Dawood MAO Koshio S Abdel-Daim MM Van Doan H. Probiotic application for sustainable aquaculture. Rev Aquac. (2018) 11:907–24. 10.1111/raq.12272

[15] Shao ZJ. Aquaculture pharmaceuticals and biologicals: current perspectives and future possibilities. Adv Drug Deliv Rev. (2001) 50:229–43. 10.1016/s0169-409x(01)00159-4 11500229

[16] Markowiak P Slizewska K. The role of probiotics, prebiotics and synbiotics in animal nutrition. Gut Pathog. (2018) 10:21. 10.1186/s13099-018-0250-0 29930711 PMC5989473

[17] Wanka KM Damerau T Costas B Krueger A Schulz C Wuertz S. Isolation and characterization of native probiotics for fish farming. BMC Microbiol. (2018) 18:119. 10.1186/s12866-018-1260-2 30236057 PMC6148792

[18] Franc A Muselik J Rabiskova M Dobiskova R. Pharmaceutical composition in the form of pellets intended for delivery to water animals and process for preparing thereof CZ308210 (B6). Available online at: https://patents.google.com/patent/CZ308210B6/en?oq=CZ308210+(B6)

[19] Hodkovicova N Hollerova A Caloudova H Blahova J Franc A Garajova M. Do foodborne polyethylene microparticles affect the health of rainbow trout (Oncorhynchus mykiss)? Sci Total Environ. (2021) 793:148490. 10.1016/j.scitotenv.2021.148490 34174619

[20] Modra H Sisperova E Blahova J Enevova V Fictum P Franc A. Elevated concentrations of T-2 toxin cause oxidative stress in the rainbow trout (Oncorhynchus mykiss). Aquac Nutr. (2018) 24:842–9. 10.1111/anu.12613

[21] Dobsikova R Blahova J Franc A Jakubik J Mikulikova I Modra H. Effect of β-1.3/1.6-D-glucan derived from oyster mushroom Pleurotus ostreatus on biometrical, haematological, biochemical, and immunological indices in rainbow trout (Oncorhynchus mykiss). Neuro-endocrinol Lett. (2012) 33:96–106. 23353851

[22] Franc A Rabiskova M Gonec R. Impregnation: a progressive method in the production of solid dosage forms with low content of poorly soluble drugs. Eur J Parenter Pharm. (2011) 16:85–91.

[23] Feckaninova A Koscova J Franc A Mudronova D Popelka P. Surviving of production probiotic strains in a selected application form | PreŽívatelnost produkčných probiotických kmenov vo vybranej aplikačnej forme. Ces slov Farm. (2022) 71:27–33. 10.5817/CSF2022-1-27 35387462

[24] Franc A Lehocky R Muselik J Vetchy D Dobiskova R Modra H. Preparation of feed premix for veterinary purposes. Ces slov Farm. (2014) 63:213–6. 25354741

[25] Ratvaj M Cingelova-Maruscakova I Schusterova P Popelka P Feckaninova A Mares J. Development of probiotic feed based on autochthonous lactobacilli and its immunomodulatory effect on rainbow trout. 11th International conference on antimicrobial agents in veterinary medicine (AAVM), 11-14.9.2022, Madrid, Spain

[26] Hollerova A Hodkovicova N Blahova J Faldyna M Franc A Pavlokova S. Polystyrene microprticles can affect the health status od freshwater fish – Threat of oral microplastic intake. Sci Total Environ. (2023) 858:159976. 10.1016/j.scitotenv.2022.159976 36347295

[27] Zeman J Pavlokova S Vetchy D Stano A Moravec Z Matejovsky L. Utilization of pharmaceutical technology methods for the development of innovative porous metasilicate pellets with a very high specific area for chemical warfare agents detection. Pharmaceutics. (2020) 13:1860. 10.3390/pharmaceutics13111860 34834274 PMC8622269

[28] Rizzelo CG Filannino P Di Cagno R Calasso M Gobbetti M. (2014). Quorum-sensing regulation of constitutive plantaricin by *Lactobacillus plantarum* strains under a model system for vegetables and fruits. Appl. Environ. Microbiol. 80: 777–87. 10.1128/AEM.03224-13 24242246 PMC3911083

[29] Refay RM Abushady HM Amer SA Mailam MA. Determination of bacteriocin-encoding genes of lactic acid bacteria isolated from traditional dairy products of Luxor province, Egypt. Future J Pharm Sci. (2020) 6:22. 10.1186/s43094-020-00031-3

[30] Tai HF Foo HL Rahim RA Loh TCH Abdullah MP Yoshinobu K. Molecular characterization of new organization of plnEF and plw loci of bacteriocin genes harbour concomitantly in Lactobacillus plantarum I-UL4. Microb Cell Fact. (2015) 14:89. 10.1186/s12934-015-0280-y 26077560 PMC4467070

[31] Omar NB Abriouel H Keleke S Valenzuela AS Martínez- Cañamero M López RL. Bacteriocin-producing Lactobacillus strains isolated from poto poto, a Congolese fermented maize product, and genetic fingerprinting of their plantaricin operons. Int. J. Food Microbiol. (2008) 127:1–2. 10.1016/j.ijfoodmicro.2008.05.037 18620772

[32] Huang Q Baum L Fu W-L. Simple and practical staining of DNA with GelRed in agarose gel electrophoresis. Clin Lab. (2010) 56:149–52. 20476647

[33] The Council of Europe European Pharmacopoeia 10th edition. Stuttgart: Deutscher Apotheker Verlag(2019).

[34] Svacinova P Vranikova B Dominik M Elbl J Pavlokova S Kubalak R. Comprehensive study of co-processed excipients F-Melts®: Flow, viscoelastic and compacts properties. Powder Technol. (2019) 355:675–87. 10.1016/j.powtec.2019.07.048v

[35] Commission regulation (EC) No 152/2009 of 27 January 2009 laying down the methods of sampling and analysis for the official control of feed. Official Journal of the European Union (2009).

[36] R Core Team. R: A Language and Environment for Statistical Computing. R Foundation for Statistical Computing. R Foundation for Statistical Computing, Vienna, Austria (2019).

[37] Pillay V Fassihi R. In vitro release modulator from crosslinked pellets for site-specific drug delivery to the gastrointestinal tract. J Control Release. (1999) 59:243–56. 10.1016/s0168-3659(98)00196-5 10332058

[38] Kuang C Bun Y Li B Fan R Zhang J Yao Y. Preparation of duloxetine hydrochlotide enteric-coated pellets with different enteric polymers. AJPS. (2017) 12:216–26. 10.1016/j.ajps.2016.08.007 32104333 PMC7032077

[39] Kareem RA Razavi SH. Plantaricin bacteriocines: as safe alternative antimicrobial peptides in food preservation-A review. J Food Saf. (2019) 40:12735. 10.1111/jfs.12735

[40] Hodkovicova N Hollerova A Blahova J Mikula P Crhanova M Karasova D. Non-steroidal anti-inflammatory drugs caused an outbreak of inflammation and oxidative stress with changes in the gut microbiota in rainbow trout (Oncorhynchus mykiss). Sci Total Environ. (2022) 849:157921. 10.1016/j.scitotenv.2022.157921 35952865

[41] Sabadkova D Franc A Muselik J Neumann D. Coated pellets with delayed release glucose for prevention of hypoglycemic episodes. Acta Pharm. (2016) 66:257–67. 10.1515/acph-2016-0017 27279068

[42] Franc A Muselik J Sabadkova D Neumann D. Preparation of pellets with controlled release of glucose as prevention of hypoglycaemia in paediatric patients. Eur J Pharm Sci. (2015) 75:72–80. 10.1016/j.ejps.2015.03.007 25805346

[43] Shabana MS Taju G Majeed A Karthika M Ramasubramanian V Sahul Hameed AS. Preparation and evaluation of mesoporous silica nanoparticles loaded quercetin against bacterial infections in *Oreochromis niloticus*. Aquac. Rep. (2021) 21:100808. 10.1016/j.aqrep.2021.100808

[44] Unban K Kanpiengjai A Khatthongngam N Saenjum C Khanongnuch C. Simultaneous bioconversion of gelatinized starchy waste from the rice noodle manufacturing process to lactic acid and maltose-forming α-amylase by Lactobacillus plantarum S21, using a low-cost medium. Fermentation. (2019) 5:32. 10.3390/fermentation5020032

[45] Gänzle MG Follador R. Metabolism of oligosaccharides and starch in lactobacilli: a review. Front Microbiol. (2012) 3:340. 10.3389/fmicb.2012.00340 23055996 PMC3458588

[46] Ghorbani S Maryam A. Encapsulation of lactic acid bacteria and Bifidobacteria using starch-sodium alginate nanofibers to enhance viability in food model. J Food Process. (2021) 45:16048. 10.1111/jfpp.16048

[47] Alfaro-Galarza O Lopez-Villegas EO Rivero-Perez N Tapia-Maruri D Jimenez-Aparicio AR Palma-Rodriguez HM. Protective effect of use of taro and rice starch as wall material on the viability of encapsulated *Lactobacillus paracasei* subsp. Paracsei. LWT. (2020) 117:108686. 10.1016/j.lwt.2019.108686

[48] Singh P Medronho B Miguel MG Esquena J. On the encapsulation and viability of probiotic bacteria in edible carbxymethyl cellulose-gelatin water-in-water emulsions. Food Hydrocoll. (2018) 75:41–50. 10.1016/j.foodhyd.2017.09.014

[49] Kiepś J Dembczyński R. Current trends in the production of probiotic formulation. Foods. 11:2330. 10.3390/foods11152330 35954096 PMC9368262

[50] Chan ES Zhang Z. Encapsulation of probiotic bacteria *Lactobacillus Acidophilus* by direct compression. Food Bioprod Process. (2002) 80:78–82. 10.1205/09603080252938708

[51] Parodi J Herrera H Sanchez R Effer B A. low-cost system forthe study of proteins used in salmonids diets, use of proteolysis to determine the quality. LWT. (2022) 165:113706. 10.1016/j.lwt.2022.113706

[52] Yus C Gracia R Larrea A Andreu V Irusta S Sebastian V. Targeted release of probiotic from enteric microparticulated formulations. Polymers. (2019) 11:1668. 10.3390/polym11101668 31614915 PMC6835770

[53] Sakandar HA Usman K Imran M. Isolation and characterization of gluten-degrading *Enterococcus mundtii* and *Wickerhamomyces anomalus*, potential probiotic strains from indigenously fermented sourdough (*Khamir*). LWT. (2018) 91:271–7. 10.1016/j.lwt.2018.01.023

[54] Todhanakasem T Ketbumrung K. Using potential lactic acid bacteria biofilms and their compounds to control biofilms of foodborne pathogens. Biotechnol Rep. (2020) 26:e00477. 10.1016/j.btre.2020.e00477 32509542 PMC7264490

[55] Noga EJ. Fish Disease: Diagnosis and Treatment. Iowa: University Press. (2010).

[56] Li D Wang Z Wu S Miao Z Du L Duan Y. Automatic recognition methods of fish feeding behavior in aquaculture: a review. Aquaculture. (2020) 528:735508. 10.1016/j.aquaculture.2020.735508

[57] Craig S Helfrich L. Understanding Fish Nutrition, Feeds, and Feeding. New York, NY: Virginia Tech. (2017), 420–256.

[58] El-Kady AA Magouz FI Mahmoud SA Abdel-Rahim MM. The effects of some commercial probiotics as water additive on water quality, fish performance, blood biochemical parameters, expression of growth and immune-related genes, and histology of Nile tilapia (*Oreochromis niloticus*). Aquaculture. (2022) 546:737249. 10.1016/j.aquaculture.2021.737249

[59] Basu S Banerjee D Chowdhury R Bhattacharya P. Controlled release of microencapsuled probiotics in food matrix. J Food Eng. (2018) 238:61–9. 10.1016/j.jfoodeng.2018.06.00522698940

[60] Cook M Tzortzis G Charalampopoulos D Khutoryanskiy VV. Microencapsulation of probiotics for gastrointestinal delivery. J Control Release. (2012) 162:56–67. 10.1016/j.jconrel.2012.06.003 22698940

[61] Fulopova N Chomova N Elbl J Mudronova D Sivulic P Pavlokova S. Preparation and evaluation of dosage form for individualized administration of lyophilized probiotics. Pharmaceutics. (2023) 15:910. 10.3390/pharmaceutics15030910 36986771 PMC10053861

[62] Melo-Bolivar JF Ruiz-Pardo RY Hume ME Sidjabat HE Villamil-Diaz LM. Probiotics for cultured freshwater fish. Microbiol Aust. (2021) 41:105–8. 10.1071/MA20026

[63] Hixson SM. Fish nutrition and current issues in aquaculture: The balance in providing safe and nutritious seafood, in an environmentally sustainable manner. J Aquac Res Dev. (2014) 5:3. 10.4172/2155-9546.1000234

[64] Maas RM Verdegem MCJ Schrama JW. Effect of non-starch polysaccharide composition and enzyme supplementation on growth performance and nutrient digestibility in Nile tilapia (*Oreochromis niloticus*). Aquac Nutr. (2019) 25:622–32. 10.1111/anu.12884

[65] Ogunkalu O. Effects of feed additives in fish feed for improvement of aquaculture. EJFST. (2019) 3:49–57.

[66] Cho CY. Fish nutrition, feeds, and feeding: With special emphasis on salmonid aquaculture. Food Rev Int. (1990) 6:333–57. 10.1080/87559129009540876

[67] Heissenberger M Watzke J Kainz MJ. Effect of nutrition on fatty acid profiles of riverine, lacustrine, and aquaculture-raised salmonids of pre-alpine habitats. Hydrobiolgia. (2010) 650:243–54. 10.1007/s10750-010-0266-z

[68] Fagbenro O Jauncey K. Physical and nutritional properties of moist fermented fish silage pellets as a protein supplement for tilapia *(Oreochromis niloticus)*. Anim. Feed Sci. Technol. (1998) 71:1–18. 10.1016/S0377-8401(97)00123-5

[69] Olmos J Acosta M Mendoza G Pitones V. Bacillus subtilis, an ideal probiotic bacterium to shrimp and fish aquaculture that increase feed digestibility, prevent microbial diseases, and avoid water pollution. Arch Microbiol. (2020) 202:427–35. 10.1007/s00203-019-01757-2 31773195

[70] Parchikolaei HM Kenari AA Esmaeilli M. Soya bean-based diets plus probiotics improve the profile of fatty acids, digestibility, intestinal microflora, growth performance and the innate immunity of beluga (*Huso huso*). Aquac Res. (2020) 52:152–66. 10.1111/are.14877

[71] Alagawany M Farag MR Abdelnour SA Elnesr SS A. review on the beneficial effect of thymol on health and production of fish. Rev Aquac. (2020) 13:632–41. 10.1111/raq.12490

[72] Ngoh SY Tan D Shen X Kathiresan P Jiang J Liew WCH. Nutrigenomic and nutritional analyses reveal the effects of pelleted feeds on Asian seabass (Lates calcifer). PLoS ONE. (2015) 10:12. 10.1371/journal.pone.0145456 26696533 PMC4687856

[73] Li X Zheng S Wu G. Nutrition and function of amino acid in fish. Amino acids in nutrition and health. Adv Exp Med Biol. (2021) 1285:133–68. 10.1007/978-3-030-54462-1_8 33770406

[74] Goel A Halami PM Tamang JP. Genome analysis of *Lactobacillus plantarum* isolated from some indian fermented foods for bacteriocin production and probiotic marker genes. Front Microbiol. (2020) 11:40. 10.3389/fmicb.2020.00040 32063893 PMC7000354

